# Effect of Chronic Pain on Fentanyl Self-Administration in Mice

**DOI:** 10.1371/journal.pone.0079239

**Published:** 2013-11-15

**Authors:** Carrie L. Wade, Perry Krumenacher, Kelley F. Kitto, Cristina D. Peterson, George L. Wilcox, Carolyn A. Fairbanks

**Affiliations:** 1 Department of Pharmacology, University of Minnesota, Minneapolis, Minnesota, United States of America; 2 Department of Neuroscience, University of Minnesota, Minneapolis, Minnesota, United States of America; 3 Department of Pharmaceutics, University of Minnesota, Minneapolis, Minnesota, United States of America; 4 Center for Pain Research, University of Minnesota, Minneapolis, Minnesota, United States of America; University of Kentucky Medical Center, United States of America

## Abstract

The development of opioid addiction in subjects with established chronic pain is an area that is poorly understood. It is critically important to clearly understand the neurobiology associated with propensity toward conversion to addiction under conditions of chronic pain. To pose the question whether the presence of chronic pain influences motivation to self-administer opioids for reward, we applied a combination of rodent models of chronic mechanical hyperalgesia and opioid self-administration. We studied fentanyl self-administration in mice under three conditions that induce chronic mechanical hyperalgesia: inflammation, peripheral nerve injury, and repeated chemotherapeutic injections. Responding for fentanyl was compared among these conditions and their respective standard controls (naïve condition, vehicle injection or sham surgery). Acquisition of fentanyl self-administration behavior was reduced or absent in all three conditions of chronic hyperalgesia relative to control mice with normal sensory thresholds. To control for potential impairment in ability to learn the lever-pressing behavior or perform the associated motor tasks, all three groups were evaluated for acquisition of food-maintained responding. In contrast to the opioid, chronic hyperalgesia did not interfere with the reinforcing effect of food. These studies indicate that the establishment of chronic hyperalgesia is associated with reduced or ablated motivation to seek opioid reward in mice.

## Introduction

Chronic pain is a broadly experienced debilitating condition that represents a significant public health concern [1]. The most effective pain relievers, opioids, are associated with risk of conversion to addiction and diversion from the patients for whom use is intended; these perceived risks constitute a recognized national health problem [1]. The mechanisms underlying the propensity for misuse and transition to opioid addiction have been studied for decades in animal models of self-administration, under normal conditions [2]. The neurobiology of addiction has been considered in the context of opioid use for treatment of chronic pain [3,4]. However, studies of opioid reward under conditions of chronic pain have been quite limited in comparison to those conducted in the normal state. This gap is significant because the neurobiology of the central nervous system (CNS) is recognized to be altered under conditions of chronic pain. 

Since the advent of animal models of chronic pain, much knowledge has been acquired regarding alterations in the central (CNS) [5-7] and peripheral (PNS) [8] nervous systems under induced states of chronic hyperalgesia. Significant differences in the reorganization of the CNS and PNS are known to occur in response to chronic pain [9,10]. More recently, progress has been made in understanding molecular changes that take place in supraspinal regions with the development of chronic pain, including regions that govern reward such as the nucleus accumbens [11], the ventral tegmental area [12-15], and the amygdala [16]. It stands to reason that, in response to chronic pain, molecular adaptations in the CNS in reward and addiction centers may impact an organism’s propensity to self-administer opioids. Several groups demonstrated differences in the consumption of opioids across a variety of pain conditions [17-22]. The goal of the present study was to determine whether chronic pain induced by diverse stimuli affects opioid self-administration. Therefore, we have evaluated the establishment of fentanyl self-administration in three distinct models of chronic hyperalgesia. Oral fentanyl responding was examined in mice with hindpaw inflammation induced by complete Freund’s adjuvant (CFA), in mice with peripheral nerve injury induced by spinal nerve ligation (SNL), and in mice with peripheral neuropathy resulting from repeated injections of the chemotherapeutic, vincristine. All experiments were conducted over a full time-course reflective of the induction and maintenance of chronic pain and included appropriate controls for chronic pain. Finally, we also evaluated lever-pressing preference for a non-opioid reinforcer, food pellets, in each of the aforementioned chronic pain conditions to examine potential learning or motor deficits.

## Materials and Methods

### Animals

All of these experiments were approved by the University of Minnesota’s Institutional Animal Care and Use Committee. Experimental subjects were Institute of Cancer Research (ICR) male mice (21-30 g, Harlan, Madison). Subjects were housed in groups of eight in a temperature- and humidity-controlled environment and maintained on a 12 hr light/dark cycle with free access water and restricted to 3 grams of food/mouse per day. 

### Chemicals

Quinine hydrochloride, CFA and vincristine sulfate were purchased from Sigma Chemical (St. Louis, MO). Fentanyl citrate was purchased from Gallipot (St. Paul, MN). CFA and vincristine sulfate were mixed in 0.9% NaCl. A concentration of 10 µg/ml was selected for fentanyl citrate based on our previous work that defined the optimal concentration [23]. Fentanyl is known to be a bitter solution [24]. Therefore, in our original dose-response analysis of oral/submucosal fentanyl we included the known bitter quinine hydrochloride (30 µg/mL) in the solution to standardize potential taste aversiveness of fentanyl solution at varying doses and in comparison to vehicle [23,25]. In order to retain uniformity across studies we continue to include quinine in the fentanyl citrate solution.

### General experimental design

**Figure 1:**
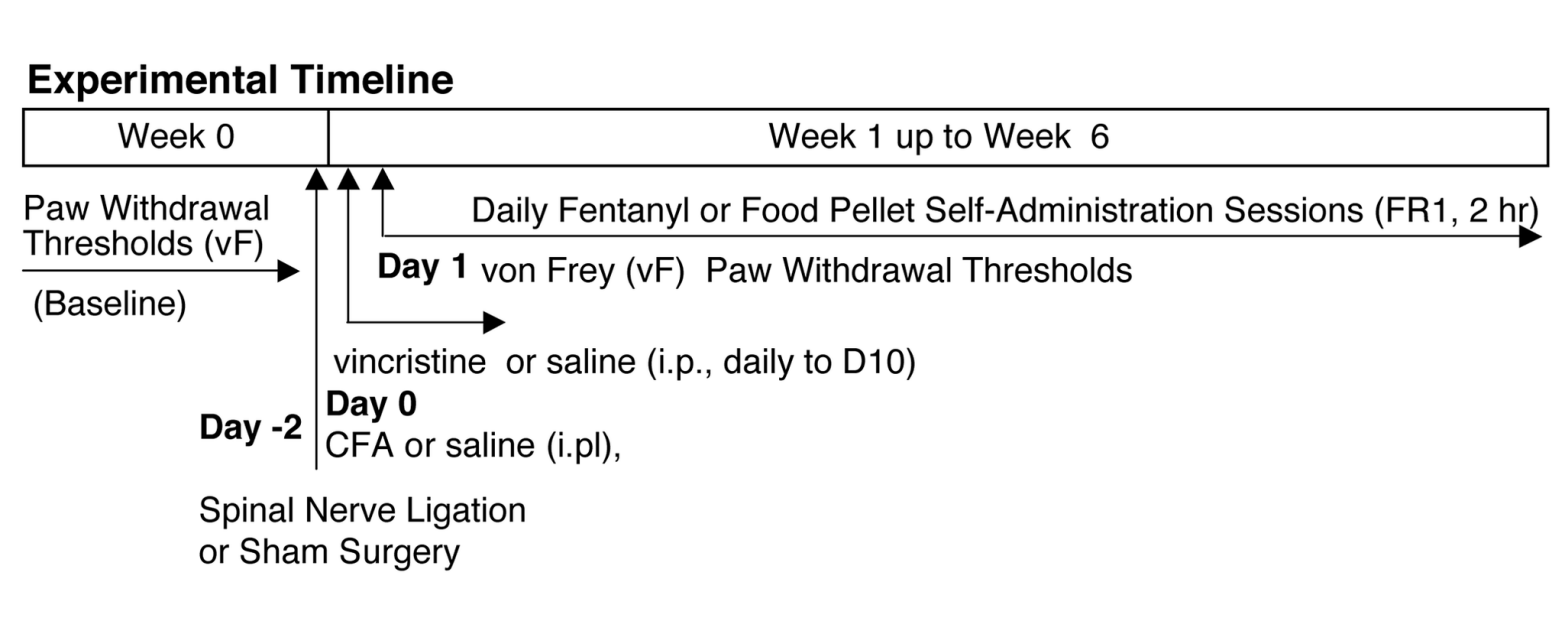
Timeline of the general experimental design.

Figure 1 illustrates the general experimental design. von Frey paw withdrawal thresholds were collected on all subjects. Hyperalgesia was induced either by intraplantar injection of CFA, spinal nerve ligation, or repeated daily injections of vincristine (through day 9). Mice were restricted to 3 grams of food/day for the duration of the study (with free access to water). On day 1 post-surgery or injection, mice began daily operant conditioning 2 hr sessions. We used the oral route of administration for fentanyl rather than i.v. catheterization because of the need to responding over a significant chronic pain time course. Oral delivery is effective for establishing fentanyl-maintained behavior in both rat [24] and mouse [23]. It is also clinically relevant because observations of oral fentanyl misuse from a variety of dosage forms are increasing [26-29]. Bar pressing on the active lever resulted in operation of the corresponding dispenser which administers 70 microliters of fentanyl solution or a food pellet where it was available for the subject to drink or consume. Bar pressing on the alternate lever (inactive) registered random activity and did not result in an outcome. These 2 hr sessions continued for a variable amount of time depending on the reinforcer (11 days for food maintained responding) and pain condition (2-6 weeks). Following a session subjects appear normal and do not exhibit overt signs of withdrawal (e.g. teeth chattering, wet-dog shakes, jumping, or abnormal behavior of any kind). Mechanical hyperalgesia was assessed periodically throughout the duration of each experiment to confirm the presence of sustained hyperalgesia. All experiments were replicated at least once and demonstrated comparable results to those data illustrated in each corresponding figure. 

### Self-administration apparatus

Experimental chambers were Modular Mouse Test Chambers (Med-Associates, St. Albans, VT). Each chamber was housed in a sound-attenuating cubicle, and equipped with a 3.33 RPM syringe pump for drug delivery, 20 mg food pellet delivery system, 2 ultra sensitive mouse levers and 2 stimulus lights. A 4.8 W house light located at the top of the cage was illuminated during experimental sessions. 

### Behavioral procedure

The FR1 reinforcement schedule coupled an active lever press with a delivery of 70 µl drug solution (or a 30 mg food pellet in control studies) to the receptacle together with illumination of the stimulus light directly above the lever. After each delivery, there was a 5 second time-out period during which fentanyl delivery was not possible, regardless of additional lever presses. Responding on the control lever resulted in illumination of the stimulus light above it, but there was no associated reinforcer provided. Responses were monitored for both the active lever and the control lever and expressed as mean responses for each test day. Each mouse was tested once daily (2 hour session) for the duration of the experiment. They were then returned to the vivarium. 

### Complete Freund’s adjuvant-induced hyperalgesia

Mice were unilaterally injected in the intraplantar region of one hindpaw with 30 microliters of a 50% solution of CFA the day before the first self-administration session.

### Spinal nerve ligation-induced neuropathic pain

Tactile hyperalgesia was induced by surgical ligation of the L5 spinal nerve as previously described for mice [30]. Mice were anesthetized with isoflurane and a mini-Goldstein retractor with a 1-cm maximum spread was then inserted into the incision at the level of the iliac crest to expose the L6 transverse process and the rostral tip of the sacrum. The L6 transverse process was then removed with use of an S&T fine forceps with a tip dimension of 0.33 x 0.25 mm (Fine Science Tools No 00108-11). Removal of the process permits visual identification of the L4-L5 spinal nerves. The L5 spinal nerve was tightly ligated with 6-0 silk thread distal to the dorsal root ganglion and proximal to the confluence of spinal nerves L4 and L5. The animals were fully mobile within 30 min of cessation of anesthetic. As a control, a separate group of animals received a sham surgery identical to the aforementioned procedure (but without nerve ligation). 

### Vincristine-induced neuropathic pain

First, mice were injected i.p. with saline, 0.03 or 0.1 of vincristine sulfate once daily for 10 days to determine optimal dose for the subsequent self-administration studies. Then, vincristine sulfate (0.1 mg/kg) was injected i.p. the day of the first self-administration session and daily for 9 days following the first injection. 

### Mechanical sensory assessment

Mice were assessed for responsiveness to mechanical stimulation using an electronic von Frey anesthesiometer (IITC Life Sciences, Woodland Hills, USA) throughout each experiment to confirm the development and persistence of mechanical hyperalgesia. Mice were placed in glass enclosures on an elevated mesh screen and allowed to acclimate for 15-30 minutes prior to sensory assessment. The electronic von Frey probe was gently applied to each hindpaw until a brisk withdrawal response terminated application of pressure (within seconds). The paw withdrawal thresholds were recorded and the force required to elicit a response was averaged over 2 applications. 

### Data Analysis

Hyperalgesia between groups was analyzed using 2-factor ANOVA. Significance was defined as p < 0.05. To assess establishment of opioid self-administration the area under the curve (AUC) for control and active levers in each treatment group was determined by the trapezoidal rule using the statistical software package JMP^®^ 6 from SAS. The resulting AUCs were analyzed using analysis of variance (ANOVA) from Prism 4.0. Significance was defined as P < 0.05. Trends were noted at p < 0.1. 

## Results

### Inflammation

**Figure 2:**
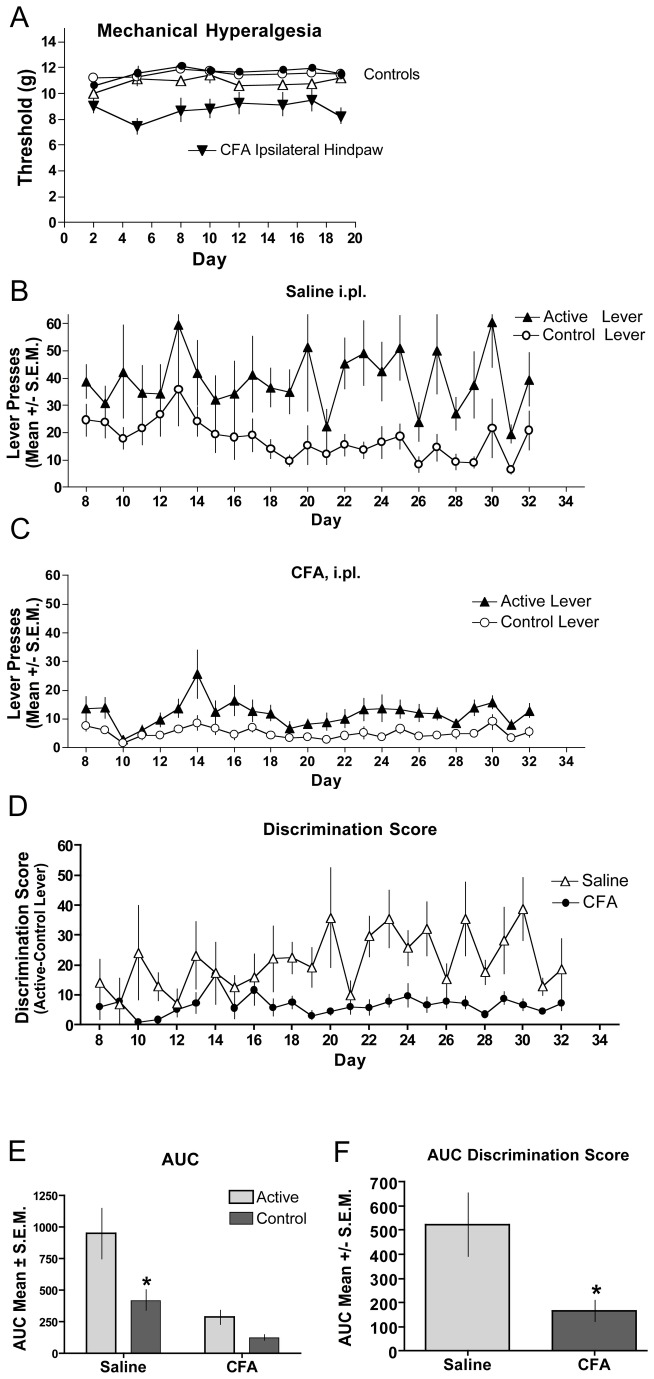
Fentanyl self-administration in CFA-induced inflammation. Complete Freund’s Adjuvant was injected intraplantarly (i.pl.) the day before the first self-administration session. (**A**) Mechanical hyperalgesia was assessed through day 19 of the study. CFA-treated hindpaws (inverted closed triangles) demonstrated significantly reduced (p < 0.05) thresholds to paw withdrawal relative to contralateral hindpaws (open triangles) or saline-treated controls (circles). Saline (**B**) or CFA (**C**) was injected i.pl. in the left hindpaw and establishment of fentanyl self-administration assessed by bar pressing on the active lever compared to the control lever. Responses represent lever presses on one of two bars. The first bar (active lever) delivers 70 µL of fentanyl (10 µg/ml) (triangles). Pressing the control lever results in no reward and is indicative of non-specific activity (circles). (**D**) The magnitude of discrimination between the active and control levers are displayed for the duration of the testing period for CFA (closed circles) and saline-treated (open triangles) mice. (**E**) Analysis of the AUC for the groups in B and C show that mice that had CFA-induced hyperalgesia did not lever press for fentanyl. (**F**) Analysis of the AUC for the groups in E show that while saline-treated mice discriminated between the active and control levers, mice with CFA-induced hyperalgesia did not. (*significance was determined by ANOVA; p < 0.05; n=8 per group).

Figure 2 shows that mice given intraplantar (i.pl.) injections of CFA show decreased paw withdrawal thresholds in the ipsilateral paw for the duration of the study, whereas the control groups (contralateral paws and saline-injected (i.pl) animals) had stable paw withdrawal thresholds throughout (Figure **2A**). Animals injected with saline (Figure **2B**) showed a significant difference in lever pressing between the active and control levers whereas mice injected with CFA (Figure **2C**) did not. To compare between groups we examined the difference score (active – control lever presses) for each daily session (Figure **2D**). An area under the curve taken from day 8-32, when stable lever pressing was achieved in the saline-injected mice, showed a significant difference in the active vs. the control levers. In contrast, the CFA-injected mice showed no difference in the active vs. the control lever (Figure **2E**). The between group analysis of the AUC resulting from the difference scores (Figure **2D**) demonstrates a significant difference between the saline- and CFA-injected mice (Figure **2F**). 

### Peripheral Nerve Injury

**Figure 3:**
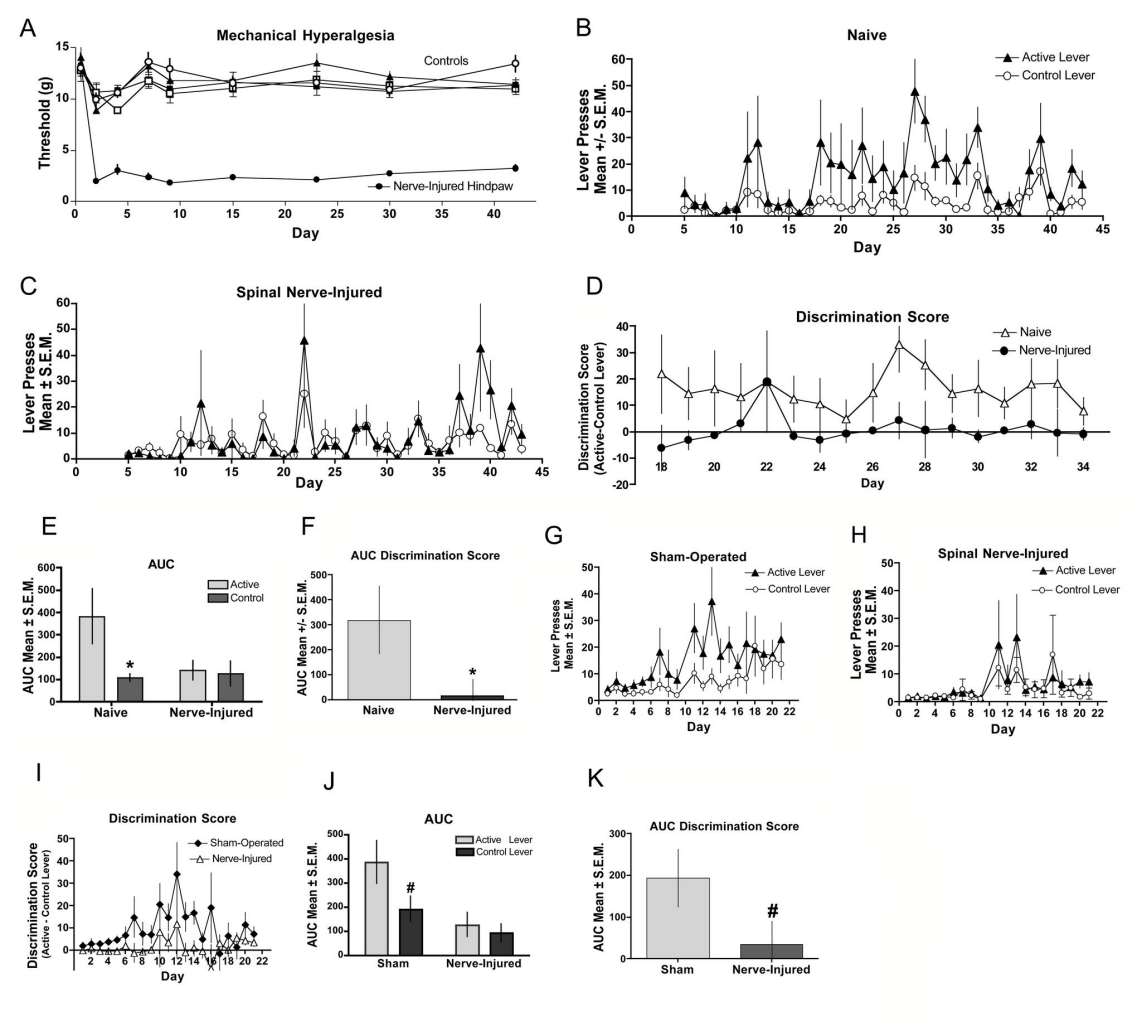
Fentanyl self-administration in peripheral nerve injury: (A-F). (**A**) Spinal nerve ligation was performed the day before the first self-administration session. Mechanical hyperalgesia was assessed through day 42 of the study. Hindpaws ispilateral to the injury (closed circles) demonstrated significantly (p < 0.05) reduced thresholds to paw withdrawal relative to contralateral hindpaws (open circles), sham-operated (triangles) or naive controls (squares). Naive (**B**) and Nerve-injured (**C**) mice were assessed for establishment of fentanyl self-administration by comparing bar pressing on the active lever with that on the control lever. (**D**) The magnitude of discrimination between the active and control levers are displayed for the duration of the testing period for naive (closed circles) and nerve-injured (open triangles) mice. (**E**) Analysis of the AUC for the groups in B and C indicate that mice with SNL-induced hyperalgesia did not lever press for fentanyl. (**F**) Analysis of the AUC for the groups in D show that naive mice discriminated between the active and control levers, while mice with SNL-induced hyperalgesia did not. (*significance for AUC was determined by Student’s t-test; p < 0.05 n=7 per group). (**G**-**K**) In a separate experiment nerve-injured mice were compared to sham-operated control mice. Sham (**G**) and nerve-injured (**H**) mice were assessed for establishment of fentanyl self-administration by comparing bar pressing on the active lever with that on the control lever. (**I**) The magnitude of discrimination between the active and control levers are displayed for the duration of the testing period for sham-operated (closed diamonds) and nerve-injured (open triangles) mice. (**J**) Analysis of the AUC for the groups in G and H show that mice with SNL-induced hyperalgesia did not lever press for fentanyl. (**K**) Analysis of the AUC for the groups in panel I show that the discrimination scores of sham-operated mice differ from that of the nerve-injured mice. # indicates a statistical trend for AUC was determined by Student’s t-test; p < 0.1, n=8 per group.

Figure 3 illustrates that mice with nerve injury showed decreased paw withdrawal thresholds in the ipsilateral paw for the duration of the study, whereas the control groups (contralateral paws, sham and naive animals) had stable paw withdrawal thresholds throughout (Figure **3A**). Responding of naive mice (Figure **3B**) on the active lever was significantly elevated relative to control lever, a difference not observed in mice with nerve injury (Figure **3C**). The difference score between active and control levers allowed for between group analysis (Figure **3D**). An area under the curve taken from day 18-34, when stable lever pressing was achieved in the naïve (control) mice, shows significant difference in responding on the active vs. the control levers for the control mice whereas the nerve-injured mice show no difference (Figure **3E**). The between group analysis of the AUC of the difference score (Figure **3D**) from days 18-34 reveals a significant difference between the naive and nerve-injured mice (Figure **3F**). In a separate experiment we compared SNL-induced nerve injury with sham-operated mice, which is the gold standard control for nerve-injured subjects. In sham-operated mice (Figure **3G**) a trend was noted for greater responding on the active lever versus control lever whereas responding in mice with nerve injury (Figure **3H, J**) was indistinguishable between the two levers. The difference score between active and control levers enabled a between-group evaluation (Figure **3I**), which indicated a trend for the sham-operated mice to have elevated AUCs in their discrimination score compared to that of nerve injured mice (Figure **3K**). 

### Vincristine-Induced Neuropathy

**Figure 4:**
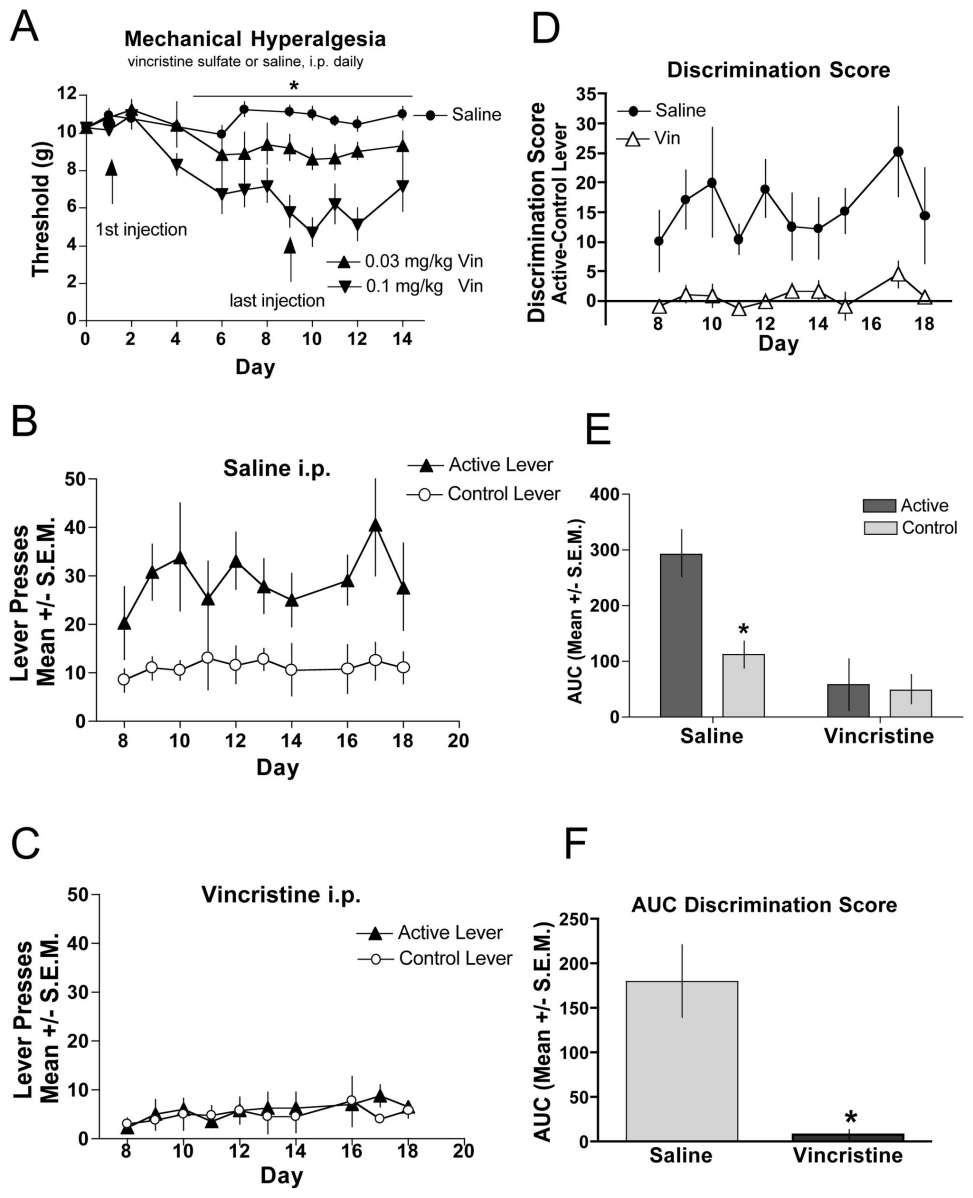
Chemotherapeutic-induced neuropathic pain. (**A**) Vincristine or saline was injected daily for 9 consecutive days. Mechanical hyperalgesia was assessed through day 14 of the study. Hindpaws of vincristine-treated subjects (triangles) demonstrated significantly (p < 0.05) reduced thresholds to paw withdrawal relative to hindpaws of saline-treated mice (circles) in a dose-related manner. N=8 per group. Saline-treated (**B**) or vincristine-treated (**C**) mice were assessed for establishment of fentanyl self-administration assessed by comparing bar pressing on the active lever with that on the control lever. (**D**) The magnitude of discrimination between the active and control levers are displayed for the duration of the testing period for vincristine (open triangles) and saline-treated (closed circles) mice. (**E**) Analysis of the AUC for the groups in B and C show that mice with vincristine-induced hyperalgesia did not lever press for fentanyl. (**F**) Analysis of the AUC for the groups in D show that the discrimination scores of saline-treated mice significantly differ between that of the vincristine-treated mice. *indicates significance for AUC was determined by ANOVA; p < 0.05, n=6 per group.

We also examined self-administration in a chemotherapy drug-induced model of neuropathic pain [31] (Figure **4**). A benefit of this model is that neuropathic pain can be induced with daily intraperitoneal (i.p.) injections and does not require invasive surgical manipulations. We first characterized the development of mechanical hyperalgesia by assessing several doses of vincristine delivery (Figure **4A**). We determined that 0.1 mg/kg resulted in the most reliable hyperalgesia and animals did not show any signs of sickness behaviors, such as decreased grooming and weight loss. In a separate population of mice that were mice chronically treated with 0.1 mg/kg vincristine (days 1-10) fentanyl self-administration was assessed for a period of 20 days. Hyperalgesia was evident by day 4 and continued for the duration of the experiment. While saline-treated controls discriminated between the active and control levers (Figure **4B**), mice with vincristine-induced neuropathic pain (Figure **4C**) did not discriminate between the levers. The discrimination scores for each group provide a direct comparison of the vincristine-injected versus saline-injected animals (Figure **4D**). An area under the curve taken from day 8-18, when stable lever pressing was achieved in the saline-injected (control) mice, demonstrate significant difference in responding on the active vs. the control levers for the control mice, whereas responding in the vincristine-injected mice indicate no lever preference (Figure **4E**). The between group analysis of the AUC defines a significant difference between the control mice and vincristine-injected mice (Figure **4F**). 

### Food-maintained responding in mice with chronic tactile hyperalgesia

**Figure 5:**
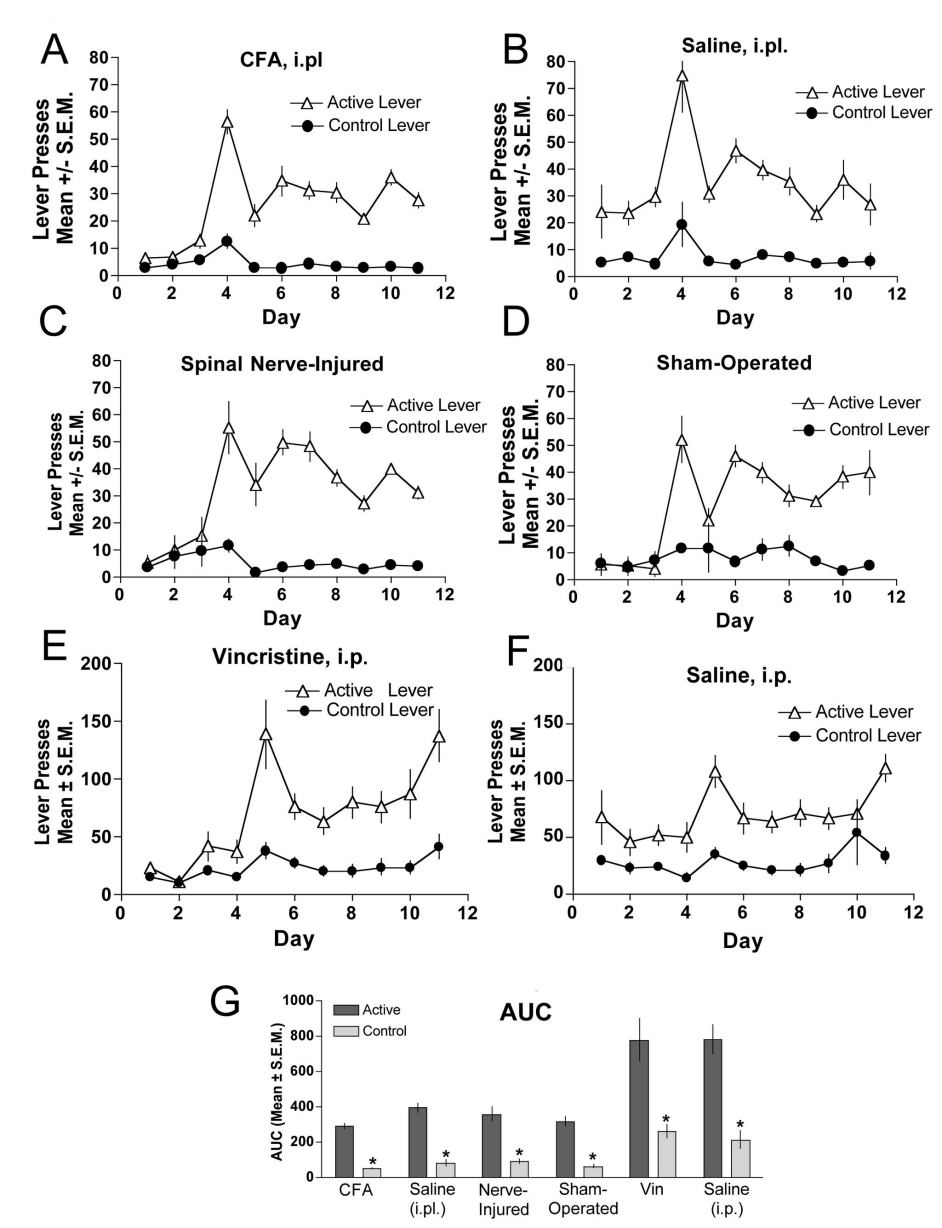
Food-maintained responding in CFA-, SNL- and vincristine-induced hyperalgesia. When compared against their respective control groups, food-maintained responding was not different between CFA-treated (**A**) versus saline-treated subjects (**B**), nerve-injured (**C**) versus sham-operated (**D**) mice, or vincristine-treated (**E**) versus saline-treated (**F**) mice. Food-maintained responding and control lever presses were recorded in daily 2 h sessions and the final area under the curve is represented in panel **G** for all groups. *indicates significant difference in responding between the respective control and active lever within experimental group was determined by ANOVA, p < 0.05., n=6 per group.

The acquisition of food-maintained responding was equivalent between mice under the induced pain conditions of CFA (Figure **5A**), SNL (Figure **5C**), or vincristine (Figure **5E**) and their respective controls (Figure **5B, D, F**) during the initial critical time period. It is shown that during the initial phase of pain induction and for out to almost 2 weeks post-induction, food-maintained responding was not changed. An area under the curve shows a significant difference between the active and inactive lever presses for all groups tested (Figure **5G**). Therefore, hyperalgesia induced by any of the manipulations used had no impact on acquisition of the operant task. These data indicate that chronic mechanical hyperalgesia results in a reduction of opioid self-administration in mice but does not affect positive reinforcers in general. 

## Discussion

Two principal observations emerge from the data presented. First, fentanyl self-administration responding was reduced or absent in three mouse models of established chronic mechanical hyperalgesia; in contrast, control mice with normal sensory thresholds acquired the behavior within several weeks. It is noteworthy that in the CFA experiment and in the second nerve injury experiment overall responding on the control lever appeared to be reduced, raising the possibility that these pain conditions may result in an overall impairment of motor activity. However, when food was introduced as a reinforcer, responding consistently developed and maintained in all groups with chronic mechanical hyperalgesia, equivalent to their respective control counterparts; this observation indicates that the lack of responding for fentanyl was not a non-specific issue related to motor function or learning deficit. These two main results demonstrate that the establishment of chronic mechanical hyperalgesia results in the reduction or elimination of the reinforcing effects of fentanyl in mouse models of chronic pain representative of inflammation, nerve injury, and chronic chemotherapeutic delivery. 

### Inflammation

Acquisition of fentanyl self-administration behavior was significantly reduced in CFA-inflamed hyperalgesic mice relative to control mice intraplantarly injected with saline. Consistent with this observation, Lyness and colleagues [18] reported that CFA-treated rats demonstrate reduced acquisition of intravenous morphine self-administration responding relative to control rats. In that study, when the assumed period of allodynia resolved, CFA-treated rats escalated their intake of intravenous morphine comparable to that of controls. Colpaert and colleagues [17] showed increased fentanyl consumption (two bottle choice model) in rats with mycobacterial inflammation, but the elevation was attenuated with non-contingent delivery of dexamethasone, indicating that the rats likely chose fentanyl for analgesic reward rather than for its other properties. That observation is consistent with the findings of Lyness et al. [18], where non-contingent subcutaneous delivery of indomethacin also further reduced morphine self-administration on day eight of the study in the CFA-treated rats only.

### Peripheral Nerve Injury

The spinal nerve ligation model [32] has been widely used to evaluate the effects of nerve injury on pain signaling and assess the potential of analgesic treatments. In this study, acquisition of fentanyl self-administration behavior was not evident in nerve-injured mice. Previously, Martin and colleagues [20] reported that nerve-injured rats demonstrated reduced responding for intravenous opioids relative to sham-operated rats, but maintained responding for food. These data were collected over a five-day period at a time of demonstrated mechanical hyperalgesia. In addition, nerve-injured animals showed rightward shifts in the ascending limb of the dose-effect curves for self-administration of morphine, fentanyl, hydromorphone, methadone, and heroin [20]. Further, this group has shown that opioid facilitation of intracranial self-stimulation in the ventral tegmental area (VTA) is depressed in spinal nerve-ligated rats, suggesting that under the condition of nerve injury, opioids may be less effective in stimulating dopamine neurotransmission [22,33]. A surprising observation of the present study was that the sham-operated subjects failed to acquire statistically significant discrimination between the active and control levers, though the fentanyl self-administration profile indicates a trend toward discrimination. Because food responding was not diminished, a likely explanation is that sham-operated mice experience post-operative pain attributable to muscle damage and bone removal during the sham operation. Gutierrez and colleagues [34] similarly noted that sham-operated subjects responded like nerve-injured subjects when self-administering a cannabinoid agonist on an FR1 schedule and speculated that incisional pain associated with the sham operation may account for these observations. Therefore, it is possible that, the post-operative pain sustained by the sham-treated group, may account for diminished magnitude of lever discrimination for fentanyl. It is, in part, this observation that provided the rationale for the study of neuropathic pain associated with chronic exposure to chemotherapeutics to enable the use of a true non-pain control group.

### Vincristine-Induced Neuropathy

Chronic chemotherapeutic-induced neuropathy was induced in mice using the vincristine model [35-38] as adapted for mice [39]. As in the other two models, acquisition of fentanyl self-administration behavior was ablated in vincristine-treated mice, but not in saline-injected control mice. In the vincristine-treated mice, responding on both levers appeared to be decreased compared to saline-treated controls during the course of the treatment period, which might raise the concern of a motor or learning deficit. As in the other two pain models, it is particularly important to note that food-maintained responding was not affected by chronic vincristine. It is also important to note that other indicators of malaise (decreased grooming, decreased weight or other factors) were not observed. von Frey filament stimulation conducted concurrently with operant conditioning training sessions confirmed that hyperalgesia was present throughout the time course studied. Although other pain models have been evaluated in operant tests of opioid self-administration, this is the first report that treatment with a chemotherapeutic agent inducing neuropathic pain reduces fentanyl self-administration. 

### Pain-Related Supraspinal Alterations

If the reinforcing profiles of opioids are reduced under the state of established chronic pain, it stands to reason that the state of chronic pain may induce alterations in the reward centers of the brain. In support of that proposal, Baliki and colleagues [40] have recently demonstrated with fMRI imaging that subjects with chronic low back pain have a different pattern of connectivity with the nucleus accumbens, the strength of which correlates with the intensity of chronic spontaneous pain. Several other reports have examined molecular alterations in reward centers in rodents with chronic opioid exposure and/or established chronic pain. Extracellular signal-related kinase (ERK) activity in the reward centers of the brain has been examined under conditions of non-contingent chronic morphine administration with and without chronic pain [12,14]. The former study demonstrated that ERK activity increased in the VTA following implantation of a morphine pellet; the observed increase in ERK activity was accompanied by an increase in tyrosine hydroxylase activity. Inhibiting ERK activity through antisense targeting ERK blocked the increase in tyrosine hydroxylase production. Ozaki et al. (2004) and Niikura and colleagues (2008) examined this phenomenon under the condition of neuropathic pain and showed that nerve-injured rats [14] and mice [15] with demonstrable thermal hyperalgesia demonstrate significantly reduced conditioned place preference when paired to morphine injection, an assay often used as a measure of reward [41]. In a separate experiment, morphine-induced conditioned place preference was also inhibited as a result of i.c.v. injection of a specific MEK inhibitor, PD98059, which blocks ERK activity [14]. In this same study, it was also demonstrated that rats with neuropathic pain have decreased ERK activity in the VTA compared to their sham control counterparts. Additionally, mice with established CFA inflammation and hindpaw carcinoma failed to demonstrate conditioned place preference to morphine [42] and showed increased binding of an anti-opioid neuropeptide FF(2) receptors in several brain regions involved in opioid reward. These results suggest that under the condition of neuropathic pain, and likely other pain conditions, morphine and other opioids, may not have the same reinforcing properties as normal control subjects. 

## Conclusion

The present study demonstrates that the condition of established chronic pain, whether induced by inflammation, peripheral nerve injury, or repeated injections of chemotherapeutics, reduces the motivation to self-administer opioids in mice. It is important to understand that this report specifically assesses the responses in subjects that have not been previously exposed to opioids. Therefore, in this study, the reduced or ablated acquisition of fentanyl self-administration in subjects with chronic pain cannot be attributable to drug-induced tolerance. Future evaluation of the propensity of chronic pain subjects to establish fentanyl self-administration following prior opioid experience will be an important next step. Such experiments will help to determine the effects of previous drug exposure on the reward system under pain conditions. Additionally, as noted previously, evidence suggests that the central nervous system at the level of the spinal cord alters in response to development and maintenance of chronic pain. Expanded evaluation of the protein and/or systems alterations that take place in the supraspinal centers associated with opioid reward in subjects with chronic pain may enable definition of the mechanism(s) by which opioid reinforcement is decreased in states of chronic pain. An understanding of how opioid and other analgesic reinforcers are experienced by subjects with established chronic pain under controlled conditions may clarify the neurobiological interaction(s) of the pain and reward systems. 
